# Physical activity, exercise and self-rated health: a population-based study from Sweden

**DOI:** 10.1186/1471-2458-8-352

**Published:** 2008-10-07

**Authors:** Marita Södergren, Jan Sundquist, Sven-Erik Johansson, Kristina Sundquist

**Affiliations:** 1 Center for Family and Community Medicine, Karolinska Institute, Stockholm, Sweden

## Abstract

**Background:**

In order to screen for the most inactive individuals in the population and target health-related interventions where they are most needed it is important to assess different forms of physical activity in population-based studies. The aims were (1) to identify the most inactive individuals in the population by assessing two dimensions of physical activity, (2) to investigate the correlation between exercise and total physical activity and (3) to investigate the association between exercise, total physical activity and good self-rated health.

**Methods:**

A simple random sample of the Swedish population aged 25–64 years were interviewed about their living conditions, health and lifestyle in a survey performed by Statitics Sweden. In total 1876 women and 1880 men completed the survey during 1999 (response rate 76.6%) when two different questions about physical activity assessed exercise and total physical activity in all domains (e.g. transportation, exercise, and at work). Logistic regression models were used to estimate odds ratios.

**Results:**

The most inactive individuals (no exercise and total physical activity ≤ 2 hours per week) constituted 4.3% of the sample. The correlation between exercise and total physical activity was low (gamma = 0.4, *p = *0.02). There were significant associations between higher levels of exercise, total physical activity and good self-rated health after adjustment for age, gender, country of birth, education, employment, marital status, housing tenure, smoking and BMI.

**Conclusion:**

Both exercise and total physical activity were independently associated with good self-rated health. It seems to be advantageous to use more than one question in population based surveys in order to evaluate several dimensions of physical activity and identify the most inactive individuals.

## Background

Physical activity has a documented positive effect on a number of health outcomes [1-7]. For example, physical activity can prevent diabetes mellitus and cardiovascular disease [1,4,6,7] and reduce mortality [5,8,9]. Previous research has also documented an association between physical activity and self-rated health[10,11]. Although self-rated health could be regarded as a subjective measure, it is a powerful predictor of morbidity and mortality [12-15]. It can also be used for cross-cultural comparisons [16].

Physical activity is often measured as physical activity during leisure-time, i.e. exercise.

Exercise is defined as "a specific type of physical activity that is planned, structured and repeatedly done to improve or maintain physical fitness, whereas the definition of physical activity is "any bodily movement produced by skeletal muscles that result in energy expenditure" [17]. The positive effects of physical activity can be gained in different ways and do not necessarily include exercise. An important limitation in studies where only exercise has been assessed is that physical activities in other domains have not been taken into account. In order to obtain a better understanding of physical activity patterns among individuals and populations it is therefore important to assess the total amount of physical activity or physical activity obtained in all domains, including exercise, physical activity at work, physical activity during transportation and household-related activities. This is particularly important in order to screen for the most inactive individuals so that health-related interventions can be targeted where they are most needed[18]. Previous studies suggest that the largest health benefits of increasing the level of physical activity can be expected among those that are most inactive [19-21].

The first aim of the present study was to identify the most inactive individuals in the population by assessing two dimensions of physical activity: exercise and total physical activity. The second aim was to investigate the correlation between exercise and total physical activity. The third aim was to investigate the association between self-rated health and the two assessments of physical activity after adjustment for the independent variables age, gender, country of birth, education, employment, marital status, housing tenure and the lifestyle factors smoking and body mass index (BMI).

## Methods

Statistics Sweden (the Swedish government-owned statistics bureau) has performed an annual survey of the adult Swedish population since 1975. Participants are interviewed face-to-face by trained interviewers about their living conditions, including questions about health and health-related conditions, welfare and lifestyle. The participants in the present study were part of that survey and consisted of a simple random sample representative of the Swedish population aged 25–64 years in 1999. Only participants from the year 1999 were included because this year was the only year when two different questions about physical activity assessed both exercise and total physical activity in all domains. The other years in the annual survey includes only one question about physical activity which is an assessment only of exercise (see below). A total of 3756 (1876 women and 1880 men) aged 25–64 completed the survey during 1999. The response rate was 76.6%. Participants aged 25–64 were selected because the focus of the present study was on individuals in working ages.

### Dependent variable

*Good self-rated health *was based on the question: "How would you describe your general health?" There were five response alternatives: "very good", "good", "fair", "poor", and "very poor". Those who answered that their general health was "very good" or "good" were considered as having good self-rated health.

### Independent variables

The present study used two survey questions to assess physical activity. The first survey question about physical activity reflected the frequency of exercise and the second question reflected the total amount of physical activity in all domains.

*Exercise *was assessed by asking the participants how often they exercise during their leisure-time and was categorized in three groups based on the following five response alternatives: (1) I get practically no exercise at all, (2) I exercise occasionally, (3) I exercise regularly, about once a week, (4) I exercise regularly, about twice a week and (5) I exercise regularly, quite vigorously at least twice a week. Those who responded to alternative 1 were categorized in group 1, those who responded to alternative 2 were categorized in group 2, and those who responded to alternatives 3, 4 or 5 were categorized in group 3. The response alternatives 3–5 were collapsed because a previous study showed that the long-term association between coronary heart disease and each of the response alternatives 3–5 was of equal size[4]. This procedure generated the following three categories for exercise: (1) None, (2) Occasionally, and (3) Regularly.

*Total physical activity *was assessed based on the following question/request: "Try to assess (during a normal week) how many hours in total you are physically active on an effort level that at least corresponds to walking. Count the total hours of physical activity, for example walking to the bus, exercise, and physical effort at work." Total physical activity in all domains of daily life (e.g. occupational, household, and exercise) was divided into three categories. The categories were based on seven response alternatives: (1) <1 hour per week, (2) 1–2 hours per week, (3) 3–5 hours per week, (4) 6–10 hours per week, (5) 11–20 hours per week, (6) 21–30 hours per week, and (7) >30 hours per week. Category 1 included response alternatives 1–2, category 2 included response alternative 3, and category 3 included response alternatives 4–7.

The choice of cut-off points resulted in three equal-sized categories for the two physical activity questions, which makes it easier to compare the categories in each group. In addition, the main focus of the present study was not on those who exercise regularly. Those individuals were therefore categorized together.

*Age *was divided into four 10-year groups: 25–34, 35–44, 45–54 and 55–64 years.

*Country of birth *was classified as Swedish-born or foreign-born.

*Educational status *was classified according to the duration of school attendance: (1) <10 years, (2) ≥ 10 years.

*Smoking habits *were divided into three groups: (1) never smokers, (2) former smokers, and (3) daily smokers.

*Body mass index *(BMI), was calculated as weight(kg)/height^2^(m), according to WHO's recommendations, and comprised three categories for both men and women: (1) normal weight (BMI < 25.0), (2) overweight (BMI 25.0–29.9) and (3) obese (BMI ≥ 30). BMI was based on self-reported weight and height. Only 1.2% of the participants were classified as being underweight (BMI < 18.5) and since they were judged not to influence the results to a large extent they were included in the normal weight category.

*Employment status *was classified as employed or nonemployed.

*Marital status *was classified as married/cohabiting or living alone.

*Housing tenure *was classified as ownership and no ownership.

These independent variables were included because previous research has shown that lifestyle and sociodemographic factors are correlated to physical activity and self-rated health [22-25].

### Statistical analyses

Logistic regression models were used to investigate the association between good self-rated health, the physical activity variables and the other independent variables. The results are presented as odds ratios (ORs) with 95% confidence interval (CI). Two models were calculated. Model 1 was adjusted for gender and age and model 2 was adjusted for all the independent variables simultaneously, i.e. gender, age, country of birth, education, smoking, BMI, employment, marital status and housing tenure. Possible interactions were tested between the two physical activity variables, each of the physical activity variables and all the other variables and gender and all the other variables. None were found. The fit of the final model was investigated by the Hosmer-Lemeshow test and was judged to be good because the p-value was higher than 0.05 (*p = *0.32). The correlation between the two physical activity questions was tested using the Goodman Kruskal gamma correlation test[26]. The statistical package used was STATA version 8 (StataCorporation, College Station TX, 2003).

### Ethical approval

This study was approved by the Ethics Committee of the Karolinska Institute, Huddinge, Sweden (reference number 11/00) and was performed in compliance with the Helsinki Declaration. Verbal informed consent was obtained from all participants.

## Results

Table 1 shows the sample size, estimated population size, mean age and percentage distribution of the individual variables. The mean age in the entire sample was 43.7 (SD 11.3) and differed only slightly between the categories.

**Table 1:** Sample size, estimated population size, mean age and percentage distribution of the individual variables.

**Variable**	**Level**	**Exercise**			**Total physical activity^**a **^(hours/week)**		
		**None**	**Occasionally**	**Regularly**	**≤ 2**	**3–5**	**≥ 6**

***Sample size * (n)**	3,756	406	1,058	2,292	538	907	2,311
***(%) ***		(10.8)	(28.2)	(61.0)	(14.3)	(24.2)	(61.5)
***Estimated population size * (N)**	4,526,437	491,396	1,279,585	2,755,456	653,043	1,095,646	2,777,748
***Mean age***	43.7	42.8	44.2	43.6	44.2	44.0	43.4
***(SD)***	(11.3)	(10.8)	(11.3)	(11.4)	(11.3)	(11.2)	(11.3)
***Gender (%)***	Women	9.9	25.6	64.5	13.8	25.2	61.0
	Men	11.8	30.7	57.5	14.8	23.2	62.0
							
***Age (%) ***	25–34 years	11.2	26.6	62.2	13.4	23.0	63.6
	35–44 years	12.6	27.7	59.7	15.4	24.0	60.6
	45–54 years	10.0	29.5	60.5	13.6	25.9	60.5
	55–64 years	9.2	29.0	61.8	15.1	23.6	61.3
***Country of birth (%)***	Swedish-born	10.1	27.6	62.3	13.6	23.8	62.5
	Foreign-born	16.1	31.9	52.0	19.5	26.3	54.2
							
***Educational status (%)***	High (≥ 10 years)	10.2	27.6	62.2	13.6	24.7	61.7
	Low (<10 years)	14.2	31.0	54.8	18.1	21.2	60.7
***Smoking habits (%)***	Never smoker	9.0	26.5	64.4	13.5	24.7	61.8
	Former smoker	9.0	27.1	63.9	13.2	24.5	62.3
	Daily smoker	17.2	33.2	49.6	17.5	22.5	60.0
***BMI***^**b **^***(%)***	Normal (<25)	9.3	25.5	65.2	11.9	24.9	63.2
	Overweight	11.4	31.3	57.3	15.7	23.9	60.4
	(25–29.9)						
	Obese (≥ 30)	17.6	32.7	49.7	23.8	20.5	55.7
							
***Employment status (%)***	Employed	10.2	28.0	61.8	13.3	23.9	62.8
	Nonemployed	13.5	29.1	57.4	19.0	25.4	55.6
***Marital status (%)***	Married/cohabiting	10.6	28.0	61.4	14.2	25.0	60.8
	Living alone	11.4	28.5	60.1	14.7	21.8	63.5
***Housing tenure (%)***	Ownership	10.2	28.8	61.0	13.7	25.0	61.3
	No ownership	12.1	26.8	61.1	15.7	22.4	61.9

n = 3756, Sweden, 1999.

^a^obtained in all domains.

^b^body mass index

Summation to 100% is done across the table for exercise and total physical activity, respectively.

Table 2 shows the distribution of the individuals by their answers to the two questions about physical activity. The most inactive individuals constituted 4.3% of the sample according to their answers to the question about exercise and the question about total physical activity in all domains. In addition, 4.9% of the individuals reported no exercise although they had ≥ 6 hours of total physical activity per week. The correlation between the two physical activity questions was rather low (gamma = 0.4, *p = *0.02).

**Table 2:** Distribution of the individuals by exercise and total physical activity.

**Total physical activity^**a **^(hours/week)**	**Exercise**		
	
	**None**	**Occasionally**	**Regularly**
**≤ 2**	160 (4.3 %)	216 (5.8 %)	162 (4.3 %)
**3–5**	62 (1.7 %)	288 (7.7 %)	557 (14.8 %)
**≥ 6**	184 (4.9 %)	554 (14.8 %)	1,573 (41.9 %)

n = 3756, Sweden, 1999.

^a ^obtained in all domains.

Table 3 shows the prevalence of good self-rated health by the physical activity categories and the independent variables. A higher prevalence of good self-rated health was found among individuals with higher levels of physical activity, higher educational level, normal weight, and individuals who were in younger ages, born in Sweden, never smokers, employed, married/cohabiting and house-owners (no statistical comparisons were performed on the data presented in Table 3).

**Table 3:** Prevalence (%) of good self-rated health by level of exercise and total physical activity and the independent variables.

**Variable**	**Level**	**Exercise**				**Total physical activity^**a **^(hours/week)**		
		**Total**	**None**	**Occasionally**	**Regularly**	**≤ 2**	**3–5**	**≥ 6**

***Total***		78.5	66.0	76.4	81.6	66.4	79.2	81.0
								
***Gender***	Women	77.6	66.0	75.4	80.2	64.1	79.2	79.9
	Men	79.4	66.1	77.2	83.3	68.5	79.1	82.1
								
***Age ***	25–34 years	85.8	69.6	83.9	89.4	76.3	86.2	87.6
	35–44 years	81.4	71.1	79.8	84.4	69.6	80.1	84.9
	45–54 years	76.1	68.0	72.4	79.2	61.5	79.8	77.7
	55–64 years	68.7	49.3	68.8	71.5	56.7	68.6	71.7
***Country of birth***	Swedish-born	80.0	70.0	77.7	82.7	68.9	81.2	82.0
	Foreign-born	67.0	48.0	68.1	72.3	53.4	65.6	72.7
								
***Educational status***	High (≥ 10 years)	81.1	69.9	78.5	84.1	69.6	81.3	83.5
	Low (<10 years)	65.1	51.7	66.8	67.6	54.1	66.2	68.0
***Smoking habits***	Never smoker	82.8	71.6	79.0	86.0	70.8	82.4	85.7
	Former smoker	77.9	63.1	78.3	79.8	70.2	78.5	79.3
	Daily smoker	69.7	61.7	69.6	72.5	54.9	72.4	73.0
***BMI***^**b**^	Normal (<25)	83.1	72.5	79.8	85.9	68.1	84.4	85.4
	Overweight (25–29.9)	76.2	66.2	75.3	78.7	71.4	75.0	78.0
	Obese (≥ 30)	59.7	46.2	64.5	61.4	50.0	59.2	64.1
								
***Employment status***	Employed	82.5	73.0	80.0	82.2	72.4	82.6	84.6
	Nonemployed	59.9	41.8	60.2	64.1	46.9	64.3	62.4
***Marital status***	Married/cohabiting	80.5	70.8	79.1	82.8	70.4	79.5	83.3
	Living alone	72.4	52.8	68.5	78.0	54.7	78.2	74.5
***Housing tenure***	Ownership	80.1	72.5	77.6	82.6	69.6	79.3	82.8
	No ownership	74.8	54.2	73.5	79.6	60.2	78.9	77.1

n = 3756, Sweden, 1999.

^a^obtained in all domains.

^b^body mass index.

Table 4 shows the ORs for good self-rated health in two models. Model 1 shows separate analyses for each independent variable and is adjusted for gender and age and model 2 is adjusted for all the independent variables simultaneously. There was an apparent gradient for both exercise and total physical activity; with increasing physical activity the odds of good self-rated health increased. The results remained statistically significant after adjustment for age, gender, country of birth, education, employment, marital status, housing tenure, smoking and BMI; however, the odds decreased. In the full model (model 2), those exercising occasionally and regularly had an OR for good self-rated health of 1.47 and 1.68, respectively. Those who reported 3–5 hours/week of total physical activity and ≥ 6 hours/week had an OR of 1.45 and 1.60, respectively, in the full model. Good self-rated health was significantly associated with all the other independent variables, with the exception of the variable housing tenure.

**Table 4:** Odds ratios (ORs) with 95% confidence intervals (CI) for good self-rated health.

**Variable**	**Level**	**Model 1**^**a**^ OR(CI)	**Model 2**^**b**^ OR(CI)
***Exercise***	None	1	1
	Occasionally	1.78 (1.38–2.29)	1.47 (1.12–1.93)
	Regularly	2.44 (1.93–3.10)	1.68 (1.29–2.18)
***Total physical activity***^**c **^***(hours/week)***	≤ 2	1	1
	3–5	1.96 (1.54–2.50)	1.45 (1.11–1.89)
	≥ 6	2.16 (1.75–2.66)	1.60 (1.26–2.02)
			
***Country of birth***	Swedish-born	1	1
	Foreign-born	0.49 (0.40–0.61)	0.68 (0.54–0.86)
***Education status***	High (≤ 10 years)	1	1
	Low (<10 years)	0.52 (0.43–0.63)	0.64 (0.52–0.79)
***Smoking habits***	Never smoker	1	1
	Former smoker	0.82 (0.68–1.00)	0.82 (0.67–1.00)
	Daily smoker	0.52 (0.43–0.64)	0.63 (0.51–0.78)
***BMI***^**d**^	Normal (<25)	1	1
	Overweight	0.70 (0.58–0.83)	0.70 (0.58–0.84)
	(25–29.9)		
	Obese (≥ 30)	0.33 (0.26–0.42)	0.40 (0.31–0.52)
***Employment ***	Employed	1	1
	Nonemployed	0.34 (0.28–0.41)	0.43 (0.35–0.52)
***Marital status***	Married/cohabiting	1	1
	Living alone	0.59 (0.49–0.70)	0.68 (0.56–0.83)
***Housing tenure***	Ownership	1	1
	No ownership	0.60 (0.50–0.71)	0.90 (0.73–1.09)

n = 3756, Sweden, 1999.

^a ^Model 1 shows separate analyses for each independent variable (nine different models) and is adjusted for age and gender.

^b ^Model 2 (full model) is adjusted for all the independent variables simultaneously. SRH, self-rated health; CI, confidence interval; OR, odds ratio.

^c ^obtained in all domains.

^d ^body mass index.

Table 5 shows the ORs for good self-rated health by the different combinations of the categories for exercise and total physical activity. Individuals that were categorized as most inactive according to both physical activity questions were used as reference. The highest odds of reporting good self-rated health was found among those that exercised on a regular basis and had a total physical activity of ≥ 6 hours/week (OR = 3.04). The combinations no exercise/total physical activity ≥ 6 hours per week and regular exercise/total physical activity ≤2 hours per week had an OR of 2.16 and 1.66, respectively. All ORs reported above are statistically significant.

**Table 5:** Odds ratios for good self-rated health by the different combinations of the categories for exercise and total physical activity, adjusted for all the independent variables simultaneously (interaction model).

**Total physical activity^**a **^(hours/week)**	**Exercise**		
	
	**None**	**Occasionally**	**Regularly**
**≤ 2**	1	1.88 (1.19–2.98)	1.66 (1.00–2.73)
**3–5**	1.21 (0.64–2.32)	2.73 (1.73–4.30)	2.55 (1.69–3.83)
**≥ 6**	2.16 (1.32–3.54)	2.22 (1.49–3.31)	3.04 (2.10–4.39)

n = 3756, Sweden, 1999.

^a ^obtained in all domains.

## Discussion

The most inactive individuals constituted only 4.3% of the sample when two dimensions of physical activity were assessed. The correlation between the two physical activity questions was low. Both exercise and total physical activity (obtained in all domains) were independently associated with good self-rated health. In addition, the highest odds of reporting good self-rated health was found among those who exercised on a regular basis and had a total physical activity of ≥ 6 hours/week.

The associations between the two forms of physical activity and good self-rated health remained significant after adjustment for several confounders, which is in accordance with previous studies[27]. In addition, the findings of the present study showed that obese individuals were less likely to rate their health as good. Lifestyle factors, including obesity, were associated with self-rated health in a recent Swedish study[24]. The association between physical activity and self-rated health has also been investigated in European countries that are part of the European Union. This study found somewhat divergent results in different countries,[10] although in the Swedish part of the study there was a positive association between physical activity and good self-rated health[10]. Other previous studies of the association between physical activity and self-rated health have also shown divergent results[11,28]. One reason for this could be that previous studies have addressed different forms of physical activities, e.g. leisure-time, occupational, household, or total physical activity. Another possible reason is that the self-assessments of health and/or physical activity are understood differently as they are subjective measures. A study from Finland and the Baltic countries showed that the association between self-rated health and physical activity varied markedly between countries, suggesting that similar associations between lifestyle and self-rated health are only found between countries with similar socioeconomic conditions[29].

In general, individuals with high socioeconomic status have higher levels of leisure-time physical activity and better self-rated health than individuals with low socioeconomic status[30]. However, the correlation between different socioeconomic variables sometimes makes it difficult to estimate the independent contribution of each of the socioeconomic variables to health-related outcomes [23]. The results of the present study showed that the socioeconomic variables education, housing tenure and employment status each were associated with self-rated health (Table 4, Model 1). This was also the case for the variable country of birth. However, the association between physical activity and self-rated health was not explained by the socio-economic factors (Table 4, Model 2).

Even if the dose-response relationship is under discussion, researchers agree that a major public health problem today is lack of physical activity[31,32]. However, the distribution of the sample by the answers to the two physical activity questions (Table 2) and the low correlation between the two questions show that it is probably not adequate to assess physical activity from a single question. If a single question is used, some individuals will be misclassified as physically inactive. The limitations of using only one physical activity question to measure physical inactivity has been discussed previously[18]. On a national level this highlights the importance of screening for the most inactive individuals in the population with more than one single question because different questions represent different dimensions of physical activity. This was demonstrated by the relatively low correlation between the two questions used in the present study.

The prevalence of Swedes that exercise more than twice a week has increased more than ten percent during the last decade, whereas the prevalence of Swedes that do not exercise has decreased only slightly[33]. Thus, a trend towards polarization can be seen, where the population is divided into those that are exercising and those that are not.

A cross-country comparison in the European Union assessed the prevalence of sedentary behaviour by the use of questionnaires in nationally representative samples [22]. Sedentary behaviour was defined in two ways: (1) those expending less than 10% of their leisure time expenditure in activities involving ≥ 4 metabolic equivalents and (2) those who did not practice any leisure-time physical activity and who also were above the median in the number of hours spent sitting down during leisure time. According to both definitions, the prevalence of sedentary people in Sweden was among the lowest in Europe. When the first and second definitions were used the prevalence rates were 43.3% and 6.4%, respectively. This suggests that the large majority of sedentary individuals in the present study were most likely found in the two lowest categories of physical activity, i.e. none and occasionally for the exercise variable and ≤ 2 hours and 3–5 hours for the total physical activity variable, i.e. about 40% of the total sample for both variables.

### Limitations and strengths

One limitation in studies of physical activity is that physical activity is difficult to measure, which limits their interpretation[34]. In addition, there is a risk of overestimation when physical activities are summarized, which could imply that some inactive individuals are classified as active. Self-report and recall bias is therefore an important limitation of the present study. Ideally, physical activity should be objectively measured. However, no such data were available in the present study. A recently published study from Sweden used objective measures and found that 52% of the individuals aged 18–69 years reached the recommended levels of physical activity [35]. Another limitation is that it cannot be excluded that some individuals do not take part in physical activity due to health problems [4]. Thus, it is difficult to assess which factors are determinants and which are consequences in the association between physical activity and self-rated health. Moreover, a recent study concluded that the association between self-rated health and exercise could be explained by predisposing genes[36]. The nature of the physical activity questions did not allow a quantification of physical activity by the different domains. For example, some studies have shown that occupational physical activity is not a predictor of good self-rated health whereas leisure-time physical activity is[28,37]. The importance of leisure-time physical activity has been reported earlier[11,27] and leisure-time physical activity is easier to influence than occupational physical activity, at least on an individual basis. In addition, the questions about physical activity did not allow assessment of irregular patterns of physical activity. On the other hand, the prevalence of irregular patterns of physical activity, so-called "weekend warriors", has proven to be relatively low[38]. Finally, our data did not allow us to assess the minimum amount of physical activity needed to improve or maintain adequate health, which is defined as either moderate-intensity physical activity for ≥ 30 min/day on ≥ 5 days/week or vigorous-intensity physical activity for ≥ 20 min/day on ≥ 3 days/week according to public health recommendations from the American College of Sports Medicine and the Centers for Disease Control and Prevention[31,32]. Moderate intensity is defined as e.g. a brisk walk[31,32]. In addition, physical activity of moderate intensity is defined as an energy expenditure of 3–6 metabolic equivalents (METs) and normal walking implies an energy expenditure of only 2.5 METs [32,39]. Our survey question assessed physical activity on an effort level that at least corresponds to normal walking, and although it is possible that the assessment also included physical activities of higher intensity we judged that our question was a better reflection of total physical activity than moderate physical activity.

One important strength of the present study was the opportunity to include two different questions about physical activity, which allowed a distinction between exercise and total physical activity (obtained in all domains) in a random sample representative of the entire Swedish population in working ages. Although this implies that the results are generalizable only among individuals aged 25–64 years, this selection was performed because several of the sociodemographic variables that were included in the study are relevant only for individuals of working age, such as employment status. The participants were interviewed face-to-face by trained interviewers and the response rate was higher than in many other similar surveys. Even after adjustment for several confounders the significant associations between physical activity and good self-rated health remained. By assessing both exercise and total physical activity in a large national survey, the present study gave a unique opportunity to examine the relationship between self-rated health and different aspects of physical activity.

## Conclusion

It is advantageous to use more than one question in order to evaluate several dimensions of physical activity and identify the most inactive individuals in the population. Population-based surveys should take this into account. Exercise and total physical activity (obtained in all domains) were independently associated with good self-rated health after adjustment for several confounders.

## Competing interests

The authors declare that they have no competing interests.

## Authors' contributions

MS participated in the design of the study, analysis and interpretation of data, drafting of the manuscript, and critical revision of the manuscript. JS participated in the design of the study, acquisition of data, analysis and interpretation of data, drafting of the manuscript, critical revision of the manuscript, obtaining funding, and supervision. SEJ participated in the design of the study, acquisition of data, analysis and interpretation of data, drafting of the manuscript, critical revision of the manuscript, and supervision. KS participated in the design of the study, analysis and interpretation of data, drafting of the manuscript, critical revision of the manuscript, obtaining funding, and supervision.  All authors read and approved the final manuscript.

## Pre-publication history

The pre-publication history for this paper can be accessed here:


